# PAR-4 overcomes chemo-resistance in breast cancer cells by antagonizing cIAP1

**DOI:** 10.1038/s41598-019-45209-9

**Published:** 2019-06-19

**Authors:** Haihong Guo, Fabian Treude, Oliver H. Krämer, Bernhard Lüscher, Jörg Hartkamp

**Affiliations:** 1 Institute of Biochemistry and Molecular Biology, Medical School, RWTH Aachen University, 52074 Aachen, Germany; 2 Department of Toxicology, University Medical Center, 55131 Mainz, Germany; 3 Present Address: Clinic for Neuropathlogy, Medical School, RWTH Aachen University, 52074 Aachen, Germany; 4 Present Address: Biofrontera Bioscience GmbH, 51377 Leverkusen, Germany

**Keywords:** Apoptosis, DNA damage response

## Abstract

Most deaths from breast cancer result from tumour recurrence, which is typically an incurable disease. Down-regulation of the pro-apoptotic tumour suppressor protein prostate apoptosis response-4 (PAR-4) is required for breast cancer recurrence and resistance to chemotherapy. Recent advances in the analysis of apoptotic signalling networks have uncovered an important role for activation of caspase-8 following DNA damage by genotoxic drugs. DNA damage induces depletion of IAP proteins and causes caspase-8 activation by promoting the formation of a cytosolic cell death complex. We demonstrate that loss of PAR-4 in triple negative breast cancer cell lines (TNBC) mediates resistance to DNA damage-induced apoptosis and prevents activation of caspase-8. Moreover, loss of PAR-4 prevents DNA damage-induced cIAP1 depletion. PAR-4 functions downstream of caspase-8 by cleavage-induced nuclear translocation of the C-terminal part and we demonstrate that nuclear translocation of the C-terminal PAR-4 fragment leads to depletion of cIAP1 and subsequent caspase-8 activation. Specifically targeting cIAP1 with RNAi or Smac mimetics (LCL161) overcomes chemo-resistance induced by loss of PAR-4 and restores caspase-8 activation. Our data identify cIAP1 as important downstream mediator of PAR-4 and we provide evidence that combining Smac mimetics and genotoxic drugs creates vulnerability for synthetic lethality in TNBC cells lacking PAR-4.

## Introduction

Whereas death rates from cardiovascular disease are steadily decreasing over the last decades, mortality rates from cancer have hardly changed and will soon surpass those from cardiovascular disease^1^. Breast cancer is the most commonly diagnosed cancer among women worldwide and is second only to lung cancer in terms of mortality^2^. Aberrant hormonal and/or growth factor signalling plays a key part in the resistance to treatment of multiple forms of breast cancer^3^. Moreover, the identification of molecular drivers has resulted in the development of highly effective forms of targeted therapy in a breast cancer subclass defined by HER2/neu amplification^4^. Despite these advances, there are currently no targeted therapies for triple negative breast cancers (TNBC) available, which are characterized only by the absence of estrogen receptor (ER), progesterone receptor (PR) and lack of amplification of the HER2/neu oncogene^5^. TNBC patients have a worse overall prognosis than other breast cancer patients although they initially respond to neoadjuvant chemotherapy^6^. From targeting individual proteins in a complex disease like TNBCs, drug development is moving towards a more system-based approach tackling dynamic oncogenic networks. In a recent study it was demonstrated that rewiring of an oncogenic signalling network by inhibiting EGFR signalling leaves TNBCs vulnerable to a second hit with DNA damaging drugs like the anthracycline Doxorubicin^7^. Mechanistically the rewiring sensitized TNBC cells to DNA damage-induced apoptosis by promoting the extrinsic apoptotic pathway, which critically requires the activation of caspase-8^7^.

The tumour suppressor protein prostate apoptosis response-4 (PAR-4) is frequently down regulated in many cancers, including renal cancer^8^, neuroblastoma^9^, endometrial carcinoma^10–12^, lung adenocarcinoma^13,14^, prostate carcinoma^15^, and breast cancer^16–19^. Most deaths from breast cancer do not result from the primary disease but from disease relapse following treatment of the primary tumour^20^. Recently, down regulation of PAR-4 in transgenic mouse models of mammary tumorigenesis and in breast cancer patients was identified as an important mechanism of tumour cell survival and recurrence at both local and distant sites following chemotherapy and targeted therapy^21^. Low PAR-4 expression independently predicts decreased recurrence-free survival in women with breast cancer, which confirms the results from previous studies^17,18^. Importantly, PAR-4 expression was also found to be low in highly aggressive, estrogen-receptor negative (ER−), basal-like, and high-grade (grade 3) breast cancers, which are all associated with poor clinical outcome^21^. These observations suggest a potential role for PAR-4 as a prognostic marker and as a drug target for breast cancer therapy.

PAR-4 is a ubiquitously expressed protein that was initially discovered as a pro-apoptotic protein in androgen-independent prostate cancer cells undergoing apoptosis in response to Ca^2+^ elevation^22^. Consistent with its pro-apoptotic activity, ectopic expression of PAR-4 sensitizes a large subset of cancer cells to apoptosis-inducing stimuli or chemotherapeutic agents^23^. Mice lacking Par-4 show reduced life span and are prone to develop spontaneous tumours in the endometrium and prostate^10^. Importantly, *in vivo* delivery of PAR-4 by adenovirus injection or nanoliposome application into tumours growing in nude mice induces tumour regression and/or tumour sensitization to therapeutic agents^24,25^.

PAR-4 consists of a unique and central SAC (Selective for Apoptosis of Cancer Cells) domain, encompassing a nuclear localisation sequence (NLS), and a C-terminal leucine zipper domain (LZ), which are both 100% conserved in human and rodent orthologous^23^. The central SAC domain has been identified by serial deletions of PAR-4 and has been described to be indispensable for the pro-apoptotic activities of PAR-4^26^. Overexpression of the SAC domain alone is sufficient to induce cell death in a variety of cancer cells but not in normal or immortalized cells^26^. Moreover, transgenic mice that ubiquitously express the SAC domain of Par-4 are resistant to the development of spontaneous as well as oncogene-induced tumours^27^.

We have previously demonstrated that UV- and TNFα-induced apoptosis results in a rapid caspase-8-dependent cleavage of PAR-4 at EEPD131/G. This process leads to nuclear accumulation of the C-terminal PAR-4 fragment that includes the SAC and LZ domains, which then induces apoptosis^28^. In the current study we investigate the influence of PAR-4 on survival of TNBC cells following genotoxic stress. We show that PAR-4 overexpression sensitizes TNBCs to genotoxic drug treatment, whereas loss of PAR-4 is accompanied with drug resistance. Furthermore, we demonstrate that in response to DNA damage PAR-4 regulates the stability of cIAP1, a member of the mammalian inhibitor of apoptosis (IAP) family, and cIAP1 antagonists can overcome chemo-resistance induced by the loss of PAR-4.

## Results

### PAR-4 expression alters drug sensitivity of TNBC cells to genotoxic stress

**Figure 1:**
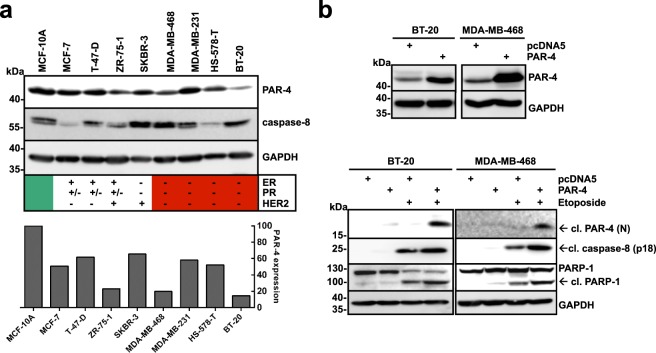
Overexpression of PAR-4 sensitizes TNBC cells to DNA damage-induced cell death. (**a**) Lysates from a panel of breast cancer cell lines including MCF-7, T-47-D, ZR-75-1, SKBR-3 and triple negative breast cancer (TNBC) cell lines MDA-MB-468, MDA-MB-231, HS-578-T and BT-20 were analysed by immunoblotting using antibodies against PAR-4, caspase-8 and GAPDH and compared with the immortalised breast epithelial cell line MCF-10A. Expression status of estrogen receptor (ER), progesterone receptor (PR) and amplification of HER2/neu (HER2) is depicted for each cell line. PAR-4 expression was also quantified with ImageJ (bottom panel). (**b**) BT-20 and MDA-MB-468 cells were transfected with control vector or PAR-4 wild-type (WT) for 24 h. Cell lysates were analysed by antibodies against PAR-4 and GAPDH. (Upper panel) Cells were transfected as in the upper panel, incubated for 24 h, stimulated with Etoposide (100 μM) for 24 h and the protein lysates were analysed with the indicated antibodies (Lower panel). (**a**,**b**) Full-length blots are presented in Supplementary Fig. S3.

**Figure 2:**
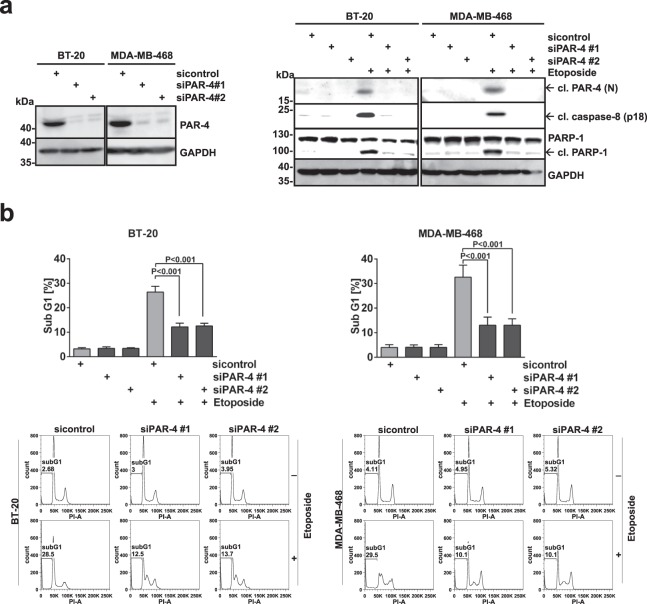
PAR-4 is required for DNA damage-induced apoptosis in TNBC cells. (**a**) PAR-4 expression was silenced in BT-20 and MDA-MB-468 cells by using two PAR-4 specific siRNAs (siPAR-4 #1 and #2), and a scrambled siRNA as a control (siControl). Knockdown efficiency was analysed 48 h after transfection. (Left panel) After 48 hours, cells were stimulated with Etoposide (100 μM) for 16 h and cell lysates were analysed by immunoblotting using the indicated antibodies. (Right panel) (**b**) BT-20 and MDA-MB-468 cells from (a) were prepared for cell cycle analysis and the sub-G1 population was measured via flow cytometry. Average values from three independent experiments are shown. Error bars indicate ± SD. *P*-value was obtained by two-tailed Student’s t-Test. (**a**) Full-length blots are presented in Supplementary Fig. S3.

As down-regulation of PAR-4 serves as a mechanism for tumour cell survival, we analysed PAR-4 expression in a panel of breast cancer cell lines by immunoblotting (Fig. 1a). Compared to the immortalized, non-transformed mammary epithelial cell line MCF-10A, none of the analysed cell lines exhibited a complete loss of PAR-4 expression. Nevertheless, PAR-4 protein levels were found to be lower in ZR-75-1 cells and in the TNBC cell lines MDA-MB-468, Hs-578T and BT-20. To further explore the function of PAR-4 in the DNA damage response (DDR) in breast cancer, the TNBC cell lines BT-20 and MDA-MB-468 were chosen for the following studies. To investigate whether PAR-4 can sensitize TNBC cells to DNA damage, wild-type (WT) PAR-4 was overexpressed in BT-20 and MDA-MB-468 cells and subsequently treated with the topoisomerase II inhibitor Etoposide (Fig. 1b). Apoptosis was analysed by caspase-8 and PARP-1 cleavage. Forced expression of PAR-4 WT alone resulted in moderate PAR-4, caspase-8 and PARP-1 cleavage in these TNBC cells. In addition, treatment with Etoposide resulted in enhanced PAR-4, caspase-8 and PARP-1 cleavage, demonstrating that overexpression of PAR-4 sensitized these cells towards DNA damage. To analyse if PAR-4 is also required for apoptosis induction following DNA damage, we silenced PAR-4 expression using siRNA and stimulated cells with Etoposide (Fig. 2a). Whereas PAR-4 cleavage was observed simultaneously to caspase-8 and PARP-1 cleavage in control cells upon Etoposide treatment, apoptosis was inhibited in PAR-4 depleted TNBC cells (Fig. 2a). Furthermore, we quantified apoptotic TNBC cells under the same conditions by measuring the sub-G1 fraction using flow cytometry. We confirmed that PAR-4 depletion led to chemo-resistance (Fig. 2b). Overall, these data demonstrate that PAR-4 sensitizes TNBC cells to genotoxic drugs and is required for DNA damage-induced apoptosis.

### Caspase-8 activity is required for DNA damage-induced cell death in TNBC cells

**Figure 3:**
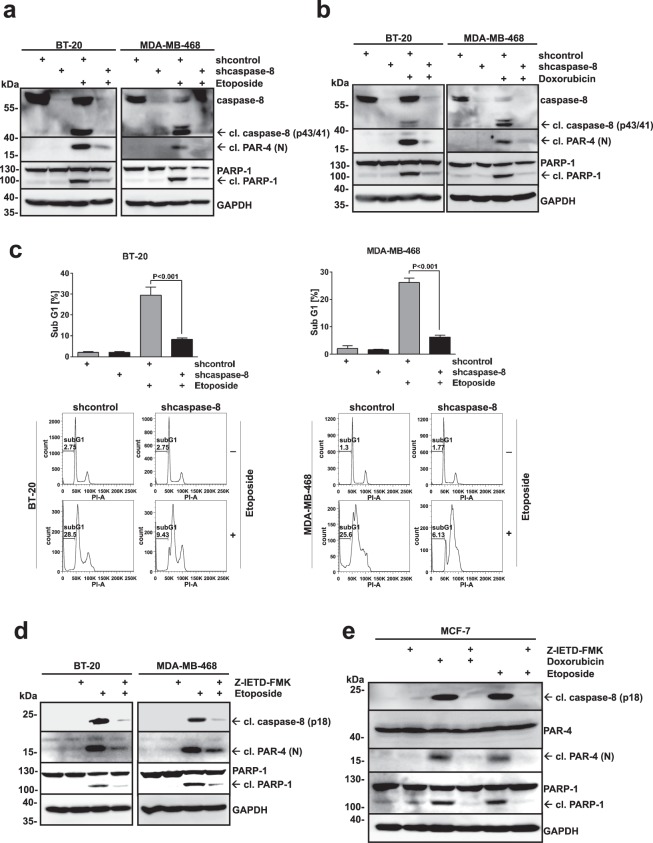
Caspase-8 activation is required for DNA damage-induced cell death. (**a**) BT-20 and MDA-MB-468 control (shcontrol) or caspase-8-deficient (shcaspase-8) cells were treated with Etoposide (100 μM) for 24 h and lysates were analysed with the indicated antibodies. (**b**) TNBC cells as in (**a**) were treated with Doxorubicin (10 μM) for 8 h and analysed as described under (**a**). (**c**) TNBCs from (**a**) were prepared for cell cycle analysis and the sub-G1 population was measured via flow cytometry. Error bars indicate ± SD. (**d**) BT-20 and MDA-MB-468 cells were pre-incubated with the caspase-8 inhibitor Z-IETD-FMK (20 μM) for 30 min followed by treatment with Etoposide (100 μM) for 24 h. Lysates were analysed via immunoblotting with the indicated antibodies. (**e**) MCF-7 cells were pre-incubated with the caspase-8 inhibitor Z-IETD-FMK (20 μM) for 30 min followed by treatment with Doxorubicin (10 μM) for 16 h or with Etoposide (100 μM) for 24 h. Lysates were analysed via immunoblotting with the indicated antibodies. (**a,b,d,e**) Full-length blots are presented in Supplementary Fig. S4.

We have previously shown that PAR-4 is a caspase-8 substrate and that caspase-8-induced PAR-4 cleavage is required for TNFα-induced cell death^28^. Recent advances in analysing apoptotic signalling networks have also uncovered an important role for activation of caspase-8 following DNA damage caused by genotoxic drugs^7,29^. For this reason we analysed whether caspase-8 activation is required for DNA damage-induced cell death in BT-20 and MDA-MB-468 cells and whether it is upstream of PAR-4 processing. Therefore, we knocked down caspase-8 expression in both cell lines using lentiviral delivery of small hairpin RNA (shRNA) constructs (Fig. 3a). Cells were treated with Etoposide or Doxorubicin (Fig. 3a,b) and analysed for caspase-8, PARP-1 and PAR-4 cleavage. The loss of caspase-8 expression led to drug resistance following DNA damage induction and also interfered with PAR-4 cleavage (Fig. 3a,b). Moreover, the knockdown of caspase-8 prevented Etoposide-induced apoptosis (Fig. 3c). In addition, pre-incubation with the caspase-8 inhibitor, Z-IETD-FMK, abolished the cleavage of PAR-4, caspase-8 and PARP-1 upon induction of DNA damage (Fig. 3d,e), indicating that caspase-8 activation is indispensable for induction of apoptosis and PAR-4 processing. Our combined data using both knock-down and inhibition of caspase-8 demonstrate that this enzyme is required for DNA damage-induced cell death and is upstream of PAR-4.

### The C-terminal PAR-4 cleavage fragment translocates into the nucleus in response to DNA damage-induced apoptosis and its expression is sufficient to induce apoptosis

**Figure 4:**
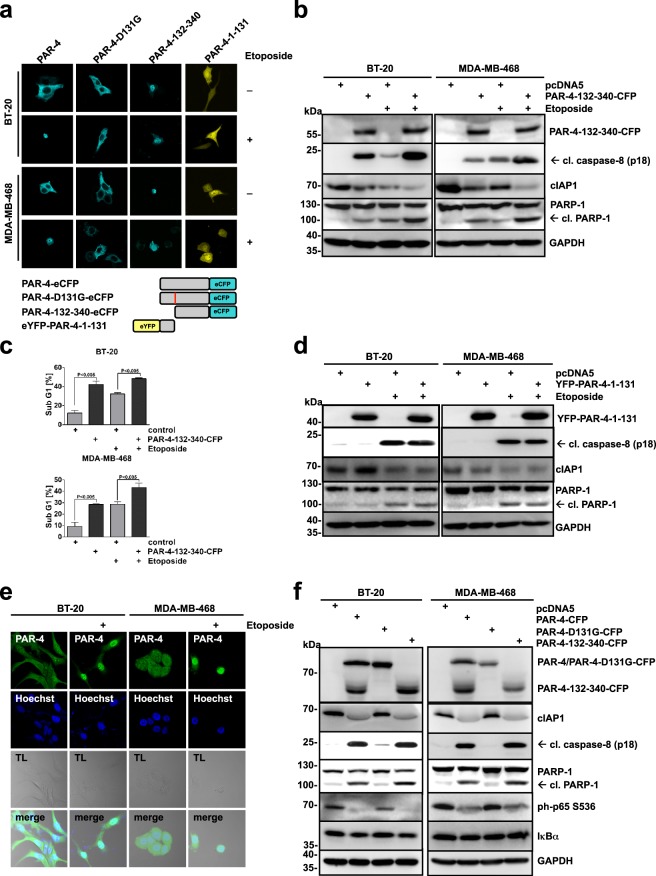
The PAR-4 C-terminal cleavage fragment translocates into the nucleus upon DNA damage-induced apoptosis and its expression alone is sufficient to induce apoptosis. (**a**) BT-20 and MDA-MB-468 cells were transiently transfected with the plasmids PAR-4-WT-eCFP, PAR-4-D131G-eCFP, PAR-4-132-340-eCFP and YFP-PAR-4-1-131 (1 μg), and after 24 h treated with Etoposide (100 μM) for an additional 24 h and analysed by immunofluorescence using confocal microscopy. Bottom panel: Scheme for PAR-4 mutants. (**b**) BT-20 and MDA-MB-468 cells were transiently transfected with 1 μg of the indicated plasmids and after 24 h treated with Etoposide (100 μM) for 16 h. Cell lysates were analysed by immunoblotting using antibodies recognizing GFP, cleaved-caspase-8, PARP-1, cIAP1 and GAPDH. (**c**) BT-20 and MDA-MB-468 were transfected and stimulated as in (**b**) and the sub-G1 population was quantified using flow cytometry. The corresponding histograms are shown in Supplementary Fig. S1. Average values from three independent experiments are displayed. Error bars indicate ± SD. *P*-value was obtained by two-tailed Student’s t-Test. (**d**) BT-20 and MDA-MB-468 cells were transiently transfected with 1 μg of the indicated plasmids and after 24 h treated with Etoposide (100 μM) for 16 h. Cell lysates were analysed with the indicated antibodies. (**e**) BT-20 and MDA-MB-468 cells were treated with Etoposide (100 μM) for 24 h followed by immunostaining with PAR-4 antibodies recognizing the C-terminal part of the protein (green). Cells were additionally treated with Hoechst for nuclear staining (blue) and analysed by confocal microscopy. (**f**) BT-20 and MDA-MB-468 cells were transiently transfected with 1 μg of the C-terminal CFP-tagged PAR-4 WT, PAR-4 D131G and the deletion mutant PAR-4 132-340 (PAR-4 132-340-eCFP) and incubated for 24 h. Cell lysates were analysed by immunoblotting using antibodies recognizing GFP, cleaved caspase-8, PARP-1 and GAPDH. (**b,d,f**) Full-length blots are presented in Supplementary Fig. S5.

We have recently demonstrated that caspase-8-mediated PAR-4 cleavage results in the nuclear accumulation of the C-terminal fragment of PAR-4 in HEK293 cells upon TNFα/CHX or UV-induced apoptosis^28^. To study the localisation of the PAR-4 cleavage products upon DNA damage in BT-20 and MBA-MD-468 cells, wild-type PAR-4, a PAR-4 cleavage resistant mutant (PAR-4-D131G), and N- and C-terminal deletion mutants (PAR-4-132-340 and PAR-4-1-131, respectively) with either a C-terminal eCFP tag or an N-terminal eYFP tag were generated and expressed in BT-20 and MDA-MB-468 cells (Fig. 4a). Whereas PAR-4-CFP and PAR-4-D131G-CFP, the latter is resistant to caspase-8 cleavage, showed cytosolic localization in both TNBC cell lines, the PAR-4 mutant lacking the amino-terminal part (PAR-4-132-340-CFP) localized to the nuclear compartment. The PAR-4 mutant lacking the C-terminal part (YFP-PAR-4-1-131) was equally distributed throughout the cell (Fig. 4a). Stimulation with Etoposide triggered a nuclear accumulation of PAR-4-CFP but was prevented in cells expressing PAR-4-D131G-CFP (Fig. 4a). In contrast, DNA damage had no influence on the localisation of the two deletion mutants. PAR-4-132-340-CFP remained nuclear and the cellular distribution of YFP-PAR-4-1-131 was unchanged (Fig. 4a). We also analysed the potential of the two PAR-4 cleavage products to induce apoptosis in TNBC cells. We observed that overexpression of the C-terminal part resulted in the activation of apoptosis, while the N-terminal region failed to do so, as documented by PARP-1 and caspase-8 cleavage as well as the accumulation of sub-G1 cells (Fig. 4b–d and Supplementary Fig. S1). To analyse whether the localisation of ectopically expressed PAR-4 behaved consistently with endogenous PAR-4, we used antibodies recognizing the C-terminal part of PAR-4 (Fig. 4e). In the absence of Etoposide, PAR-4 mainly localized to the cytosolic compartment, whereas the staining accumulated in the nucleus after Etoposide treatment, confirming that the overexpressed PAR-4 behaves comparable to the endogenous protein (Fig. 4e). Given that the cleaved C-terminal fragment, which contains the SAC domain, is sufficient to induce apoptosis in cancer cells (Fig. 4b,c)^24^, we investigated whether overexpression of the PAR-4 cleavage resistant mutant was capable to evoke apoptosis in BT-20 and MDA-MB-468 cells. The pro-apoptotic properties of PAR-4 involve inhibition of the NFκB pathway^30^. Indeed, PAR-4-D131G was unable to inhibit NFκB activity and induce apoptosis, in contrast to PAR-4 and PAR-4-131-340 (Fig. 4f). Together, these findings are in line with a pro-apoptotic function of the C-terminal PAR-4 fragment after localisation to the nuclear compartment.

### PAR-4 regulates cIAP1 depletion upon DNA damage-induced apoptosis

**Figure 5:**
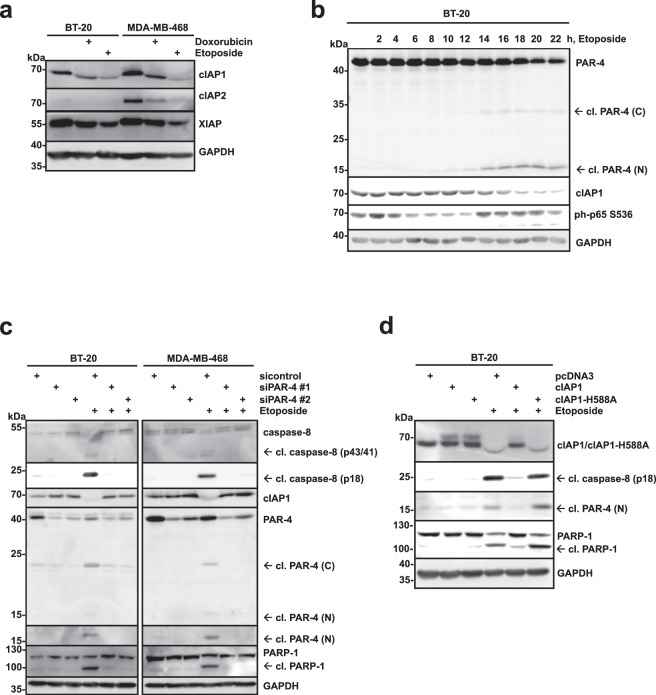
PAR-4 regulates cIAP1 depletion upon DNA damage-induced apoptosis. (**a**) BT-20 and MDA-MB-468 cells were stimulated with Doxorubicin (10 μM) for 8 h, or Etoposide (100 μM) for 16 h and cell lysates were analysed by immunoblotting using the indicated antibodies. (**b**) BT-20 cells were treated with Etoposide (100 μM) for the indicated time points and cell lysates were analysed by immunoblotting using specific antibodies for PAR-4, cIAP1, ph p65 Ser536 and GAPDH as control. (**c**) Par-4 expression in the indicated TNBC cells was silenced as shown in (Fig. 2a) and stimulated with Etoposide (100 μM) for 16 h. Cell lysates were analysed by immunoblotting using antibodies specific for PAR-4, PARP-1, cleaved caspase-8 and GAPDH. (**d**) BT-20 cells were transiently transfected with myc-tagged cIAP1 WT, cIAP1 H588S mutant for 24 h, and were indicated, cells were treated with Etoposide (100 μM) for 16 h. Cell lysates were analysed by immunoblotting using the indicated antibodies. (**a**–**d**) Full-length blots are presented in Supplementary Fig. S6.

Our data so far indicates, that loss of PAR-4 interferes with the activation of caspase-8 in breast cancer cells after stimulation with Etoposide (Fig. 2a). Genotoxic stress induces the depletion of cIAP1, cIAP2 and XIAP, which triggers the assembly of a RIP-1/FADD/caspase-8 complex and can initiate autocatalysis and activation of caspase-8^31^. We noticed in our experiments a reduced expression of cIAP1 in response to transiently expressed PAR-4 and the C-terminal PAR-4 fragment (Fig. 4b,d,f). To address the involvement of IAP proteins more broadly, the expression of cIAP1, cIAP2 and XIAP, was analysed in BT-20 and MDA-MB-468 cells in response to treatment with Doxorubicin or Etoposide (Fig. 5a). We found that cIAP1 and XIAP were expressed in both BT-20 and MDA-MB-468 cells, while cIAP2 was only detectable in the latter. All three IAPs were reduced to different degrees in response to DNA damage (Fig. 5a). As we had observed before that the C-terminal PAR-4 fragment was sufficient to deplete cIAP1 in TNBCs (Fig. 4) and because cIAP1 was efficiently depleted after cytotoxic drug treatment (Fig. 5a), we focused on the correlation of PAR-4 and cIAP1. First, we analysed whether PAR-4 cleavage correlated with cIAP1 depletion in a time-dependent manner. BT-20 cells were stimulated with Etoposide for increasing length of time (Fig. 5b). PAR-4 cleavage was detectable after 12–14 hours (Fig. 5b). The appearance of cleaved PAR-4 correlated with a decrease in cIAP-1 expression while at earlier time points no effect of Etoposide on cIAP1 could be measured (Fig. 5b). To analyse whether PAR-4 was required for DNA damage-induced cIAP1 depletion, we compared PAR-4-deficient cells with control cells after Etoposide treatment. Whereas caspase-8 activation and cIAP1 depletion was efficiently induced in control TNBCs, it was significantly inhibited in PAR-4-depleted cells (Fig. 5c). To determine whether cIAP1 overexpression was capable to protect BT-20 cells from apoptosis and caspase-8 activation, we overexpressed cIAP1 WT and a catalytic inactive cIAP1-H588A mutant and stimulated the cells with Etoposide. Only the wild-type cIAP1 was able to inhibit induction of apoptosis, caspase-8 activation and PAR-4 cleavage, whereas the catalytically inactive mutant failed to do so (Fig. 5d). In summary, these data suggested that PAR-4 expression is required to induce cIAP1 depletion in response to DNA damage and subsequent caspase-8 activation. Furthermore, ectopic expression of cIAP1 interferes with these processes.

### Loss of cIAP1 sensitizes TNBCs to DNA damage-induced apoptosis when PAR-4 expression is depleted

**Figure 6:**
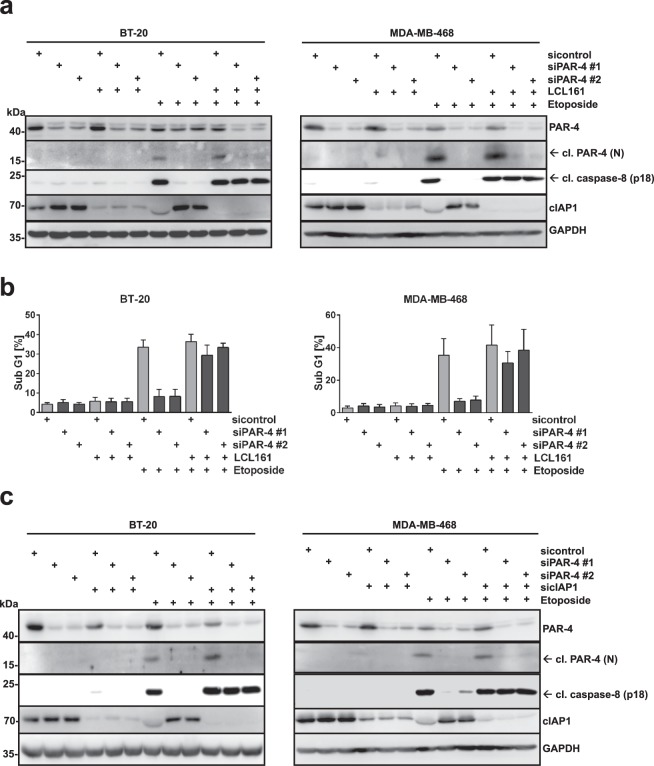
Depletion of cIAP1 using Smac Mimetic (LC161) or siRNA, sensitizes TNBCs to DNA damage-induced apoptosis in drug resistant PAR-4 deficient cells. (**a**) Par-4 expression in the indicated TNBC cells was silenced as shown in (Fig. 2a). Cells were pre-treated with cIAP1 antagonist LCL161 (0,5 μM) for 1 h and then stimulated with Etoposide (100 μM) for 16 h. Cell lysates were analysed by immunoblotting using the indicated antibodies. (**b**) TNBCs from (**a**) were prepared for cell cycle analysis and the sub-G1 population was measured via flow cytometry. The corresponding histograms are presented in Supplementary Fig. S2. Average values from three independent experiments are shown. Error bars indicate ± SD. (**c**) PAR-4 and cIAP1 expression were silenced in BT-20 and MDA-MB-468 cells by using specific siRNAs (siPAR-4 #1, siPAR-4 #2 and sicIAP1). After incubation for 48 h cells were stimulated with Etoposide (100 μM) for 16 h and cell lysates were analysed by immunoblotting using the indicated antibodies. (**a**,**c**) Full-length blots are presented in Supplementary Fig. S7.

The expression of IAP proteins, including cIAP1, is frequently altered in various human cancers and its expression is often associated with disease progression and poor prognosis^32,33^. Consequently, IAP proteins are considered as candidate drug targets for therapeutic intervention. Small-molecule inhibitors, referred to as Smac (second mitochondrial-derived activator of caspases) mimetics (SM), have been developed to target IAPs and are currently undergoing clinical trials for cancer treatment^34,35^. As loss of PAR-4 prevented cIAP1 depletion and induction of apoptosis after genotoxic drug treatment (Fig. 5c), we analysed whether depletion of cIAP1 could revert this effect. In agreement with previous reports, treatment of TNBCs with the Smac mimetic LCL161, which specifically promotes degradation of cIAP1 and cIAP2^36,37^, resulted in cIAP1 depletion (Fig. 6a). We observed that pre-treatment of PAR-4 depleted TNBCs with SM led to an efficient activation of caspase-8 and induction of apoptosis after Etoposide treatment, strongly suggesting that the chemo-resistance induced by PAR-4 deficiency can be rescued by cIAP1 antagonists (Fig. 6a). This was further confirmed by measuring apoptotic cell death in BT-20 and MDA-MB-468 cells using flow cytometry (Fig. 6b and Supplementary Fig. S2). To verify these data we depleted cIAP1 expression using cIAP1-specific siRNA and confirmed that the loss of cIAP1 in PAR-4 deficient and drug resistant TNBCs sensitized both cell lines to DNA damage-induced apoptosis (Fig. 6c). Together, these data indicate that PAR-4 sensitizes TNBC cells to DNA damage via regulation of cIAP1 degradation.

## Discussion

TNBCs are a heterogeneous mix of breast cancers defined by lack of ER and PR expression and absence of amplification of the HER2/neu oncogene. Therefore, patients with triple-negative breast cancer do not benefit from hormonal or trastuzumab-based therapies and standard treatment includes mainly DNA damaging chemotherapy^38^. Although TNBC patients are reported to initially respond to genotoxic chemotherapy, the overall survival of these patients is still poor^39^. Thus, identifying new strategies to overcome drug resistance of TNBC cells may have a therapeutic benefit.

In a recent study the pro-apoptotic tumour suppressor protein PAR-4 was identified as a critical negative regulator of residual cell survival and recurrence in mice and humans^21^. Furthermore, low PAR-4 expression was found to be associated with a poor response to genotoxic chemotherapy and an increased risk for disease relapse in breast cancer patients^21^. Therefore, therapies that either target PAR-4 effector pathways or restore PAR-4 expression may be promising for therapeutic intervention. Interestingly, PAR-4 is transcriptionally repressed by activation of the PI3K-AKT-mTOR signalling pathway and forkhead transcription factors like Foxo3a were identified to mediate this transcriptional regulation of PAR-4^40^. Importantly, Foxo-dependent upregulation of PAR-4 was found to prevent cell survival following treatment with drugs targeting the PI3K-AKT pathway^40^.

In the present study we have elucidated the effector pathways regulated by PAR-4 in response to DNA damaging chemotherapy in two triple negative breast cancer cell lines. We found that loss of PAR-4 interferes with DNA damage-induced depletion of cIAP1 and subsequent caspase-8 activation. Moreover, targeting cIAP1 with Smac mimetics or RNA interference reverted drug resistance induced by the loss of PAR-4 in TNBC cells.

**Figure 7:**
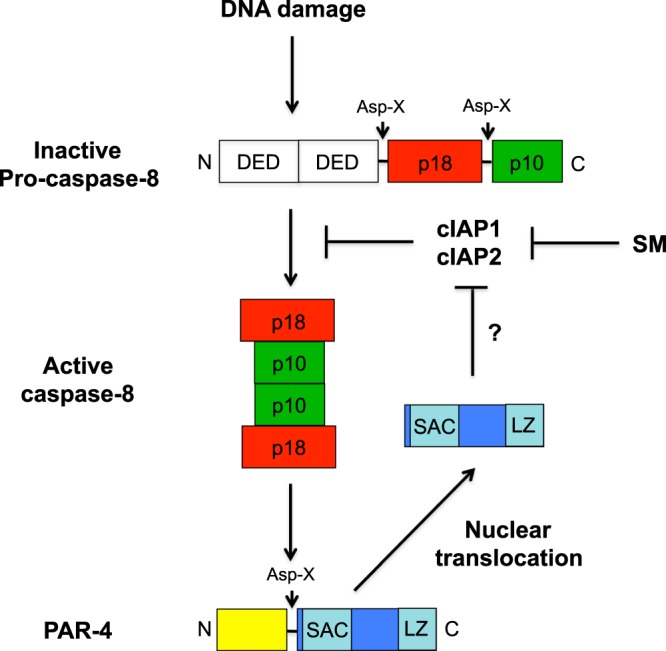
Schematic illustration: PAR-4-induced activation of caspase-8 following DNA damage is mediated by controlling cIAP1. Abbreviation: Smac mimetic (SM).

We and others have previously reported that PAR-4 is a caspase target^28,41–43^. Our studies have demonstrated that caspase-8 cleaves PAR-4 *in vitro* and mechanistically we have reported that TNFα-induced apoptosis led to caspase-8-mediated PAR-4 cleavage followed by the nuclear accumulation of the C-terminal PAR-4 fragment, which then induces cell death^28^. Although caspase-8 activation is generally thought to be specific to receptor-mediated apoptosis, sequential application of EGFR inhibitors, combined with DNA-damaging chemotherapy has shown before to strongly enhance caspase-8 activation and subsequent cell death in TNBCs^7^. In agreement with this, treatment of TNBCs with the topoisomerase II inhibitors Doxorubicin or Etoposide resulted in an efficient activation of caspase-8 (Fig. 3a,b). Moreover, cleavage of PAR-4 coincided with PARP-1 cleavage, suggesting that DNA damage also triggers caspase-8-mediated PAR-4 cleavage (Fig. 3). Indeed, loss of caspase-8 expression or the inhibition of its activity in BT-20 and MDA-MB-468 demonstrate that DNA damage-induced cell death and PAR-4 cleavage is caspase-8 dependent (Fig. 3). Furthermore, DNA damage-induced PAR-4 cleavage in caspase-3-deficient MCF-7 cells was also sensitive to caspase-8 inhibition and hence PAR-4 functions downstream of caspase-8 (Figs 3e and 7).

To analyse the influence of PAR-4 in the DDR we depleted PAR-4 in BT-20 and MDA-MB-468 cells (Fig. 2a). Our results demonstrate that loss of PAR-4 abrogated DNA damage-induced cell death and surprisingly also prevented caspase-8 activation, indicating that PAR-4 interferes with the activation of the initiator caspase-8 via an unknown feedback mechanism (Fig. 2a). Recent evidence indicates that mammalian inhibitor of apoptosis (IAP) protein family members play a key role in regulating the ripoptosome, a novel cell death-inducing platform that includes caspase-8^31,44^. The ripoptosome assembles in response to DNA damage-induced depletion of XIAP, cIAP1 and cIAP2 and can stimulate caspase-8-mediated apoptosis^31^. A negative correlation between PAR-4 and XIAP has already been observed in *Par-4* knockout mice, where Xiap levels were enhanced in primary mouse embryo fibroblasts and in the uteri of female mice^12,30^. Indeed, DNA damage resulted in depletion of XIAP, cIAP1 and cIAP2 in TNBCs with the strongest effect on cIAP1 (Fig. 5a). A time course analysis after induction of genotoxic stress confirmed simultaneous PAR-4 cleavage and cIAP1 depletion (Fig. 5b). Moreover, ectopic expression of cIAP1 prevented genotoxic stress-induced caspase-8 activation, whereas a cIAP1 inactive mutant failed to do so (Fig. 5d). Importantly, loss of PAR-4 completely blocked DNA damage-induced depletion of cIAP1, which was required for genotoxic stress-induced caspase-8 activation (Fig. 5c). DNA damage induced the cleavage of PAR-4 and only the C-terminal fragment translocated into the nucleus where it induced cell death, whereas a cleavage resistant mutant failed to do so (Fig. 4). Only the C-terminal part induced depletion of cIAP-1 and simultaneous caspase-8 activation in TNBC cells (Fig. 4f). Hence caspase-8-mediated PAR-4 cleavage resulted in nuclear translocation of the C-terminal part and induced cIAP1 depletion by an unknown mechanism, which is required for full activation of caspase-8.

Finally, we analysed whether inhibition of cIAP1 can overcome the resistance to genotoxic stress induced by the loss of PAR-4 (Fig. 6). Smac mimetics are small-molecule inhibitors that mimic Smac, an endogenous antagonist of cIAP1, cIAP2 and XIAP. The interaction of Smac mimetics with cIAP proteins promotes their autoubiquitination and proteasomal degradation^34,35^. Indeed, depleting cIAP1 with the Smac mimetic compound LCL161 completely abrogated DNA damage-induced resistance mediated through the loss of PAR-4 (Fig. 6a,b). Furthermore, silencing cIAP1 with RNAi in TNBCs confirmed the specificity for cIAP1 as an important mediator in genotoxic stress-induced resistance as a consequence of down regulation of PAR-4 (Fig. 6c). Currently, several Smac mimetics are under evaluation in clinical trials either in monotherapy or in combination with cytotoxic therapies^34,35^.

In summary, our data suggest an important role for caspase-8 in the execution of cell death in response to DNA-damaging chemotherapy in TNBC. This is also supported by the fact that *CASP8* truncation mutations were identified in a large screen aiming to identify somatic driver mutations in breast cancer^45^. Caspase-8-induced PAR-4 cleavage results in the nuclear accumulation of the C-terminal fragment, which induces cIAP1 depletion and thereby facilitates full activation of caspase-8 in a feedback loop (Fig. 7). Moreover, loss of PAR-4 in TNBCs completely prevents cIAP1 depletion induced by genotoxic stress and results in drug resistance. Importantly, targeting cIAP-1 with Smac mimetics in TNBC overcomes chemo-resistance induced by down regulation of PAR-4. Together, this suggests that tumour cells with low PAR-4 expression might be particularly vulnerable to Smac mimetics combined with chemotherapy. As low PAR-4 expression is associated with poor response to neoadjuvant chemotherapy and an increased risk of relapse in breast cancer patients^21^, combining Smac mimetics with chemotherapy might be a promising treatment option for these patients.

## Methods

### Cell lines, cell culture and transfections

All cell lines were cultured in a humidified atmosphere at 37 °C with 5% CO_2_. MDA-MB-468, MCF-10A and Hs 578T cells were cultivated in DMEM, while BT-20, MCF-7, MDA-MB-231, T-47D and ZR-75-1 cells were kept in RPMI-1640 medium, and SK-BR-3 cells were cultivated in McCoy’s 5 A medium. All media were supplemented with 10% heat-inactivated foetal calf serum (FCS). To achieve a stable Caspase-8 knockdown the following plasmids from the Thermo Scientific GIPZ lentiviral shRNA library were used: Caspase-8 (V2LHS_112733; referred to as shCaspase-8) or non-silencing control (RHS4346; referred to as shControl). Lentiviral transduction procedures were carried out as described before^28^, and 2 μg/ml puromycin (Sigma) to the medium of BT-20 or MDA-MB-468 cells. Transient transfections of BT-20 or MDA-MB-468 cells was carried out by using Lipofectamine® 2000 (Invitrogen) according to the manufacturer’s instructions. A transient knockdown of PAR-4 was achieved using two pre-designed siRNAs constructs directed against human PAR-4 (SI02628997 and SI03071782; referred to as siPAR-4 #1 and siPAR-4 #2) at a final concentration of 20 nM and compared to a non-silencing siRNA (SI03650325; referred to as siControl) (Qiagen). A knockdown of cIAP1 was achieved using siRNA constructs directed against human cIAP-1 (sc-29848; referred to as sicIAP1) (Santa Cruz). Transfection was achieved using Oligofectamine^TM^ (Invitrogen) transfection reagent according to the supplier’s instruction.

### Reagents and antibodies

The following reagents were used: Doxorubicin (Sigma), Etoposide (Sigma), Z-IETD-FMK (Santa Cruz), LCL161 (Selleckchem), anti-PAR-4 (sc-1807, Santa Cruz), anti-PAR-4 (ab5787, Abcam), anti-GAPDH (sc-32233, Santa Cruz), anti-caspase-8 (#9746, Cell Signaling), anti-cleaved caspase-8 (#9496, Cell Signaling), anti-PARP-1 (#9542, Cell Signaling), anti-cIAP1 (#7065, Cell Signaling), anti-cIAP2 (#3130, Cell Signaling), anti-XIAP (#2045, Cell Signaling), anti-phospho-p65 Ser536 (#3033, Cell Signaling), anti-IκBα (#9242, Cell Signaling), anti-GFP (Rockland), anti-rabbit-HRP (P0448, DAKO), anti-mouse-HRP (P0447, DAKO), anti-rabbit-Alexa-Fluor-555 (A-31572, Thermo Fisher) and anti-rabbit-Alexa-Fluor-488 (A-21206, Thermo Fisher).

### Cloning and mutagenesis

Generation of pcDNA5/FRT/TO-PAR-4(132-340)-eCFP, pcDNA5/FRT/TO-PAR-4wt-eCFP, pcDNA5/FRT/TO-PAR-4(D131G)-eCFP and pcDNA5/FRT/TO-eYFP-PAR-4(1-131) was described before^28^. Plasmids for pcDNA3-myc-cIAP1 and pcDNA3-myc-cIAP1 mutant (H588A) were provided by S. Schreek.

### Immunofluorescence microscopy

BT-20 and MDA-MB-468 cells were transiently transfected with plasmids encoding PAR-4 WT-eCFP, PAR-4 D131G-eCFP, PAR-4 (132-340)-eCFP and eYFP-PAR-4 (1-131) for 24 h, washed with PBS and fixed with 3.7% (w/v) paraformaldehyde (PFA)/PBS for 20 min. Cells were permeabilized with PBS containing 0,1% Triton-X-100 for 30 min and blocked in 3% bovine serum albumin (BSA) in PBS for 1 h. Endogenous PAR-4 was stained with PAR-4-specific antibodies (Abcam, 1:100) and visualized with secondary Alexa Fluor 555 conjugated antibodies (1:1000). Hoechst 33258 (2 μg/ml, Sigma) was added and coverslips were mounted with Immu-Mount (Thermo Fisher). Image visualisation was performed with the Zeiss LSM 710 confocal microscope using a LDC-apochromat 40×/1.1 water objective. ZEN 2009 (Zeiss) software was used for image editing.

### Flow cytometry analysis

TNBCs were washed once in PBS and fixed with 80% (v/v) ethanol, stored at −20 °C, for 30 min. After fixation, cells were resuspended in PBS and to avoid unspecific staining of RNA DNAse free RNAse (20 μg/ml, Roche) was added and incubated for 5 min at RT. Finally, propidium iodide (Sigma) was applied to a final concentration of 50 μg/ml and cells were incubated protected from light for 20 min at RT. Cell cycle analysis was performed using the BD FACSCanto II flow cytometer. Flowing software version 2.4.1. was used for data editing. Flow cytometry data was analysed using FlowJo software (V.8.8.6, Tree Star, Ashland, Oregon, USA).

## Supplementary information

PAR-4 overcomes chemo-resistance in breast cancer cells by antagonizing cIAP1
