# Supplementary material for: PAR-4 overcomes chemo-resistance in breast cancer cells by antagonizing cIAP1
Source: Sci Rep. 2019 Jun 19;9:8755. doi: 10.1038/s41598-019-45209-9 (PMC6584570; doi:10.1038/s41598-019-45209-9)
Supplement: Supplementary file 1 — PAR-4 overcomes chemo-resistance in breast cancer cells by antagonizing cIAP1 [file 41598_2019_45209_MOESM1_ESM.pdf]

Supplementary Information

PAR-4 overcomes chemo-resistance in breast cancer cells by antagonizing cIAP1

Haihong Guo[1,3,#], Fabian Treude[1,#], Oliver H. Krämer[2], Bernhard Lüscher[1], Jörg Hartkamp[1,3,*]

[1]Institute of Biochemistry and Molecular Biology, Medical School, RWTH Aachen University, 52074 Aachen, Germany

[2]Department of Toxicology, University Medical Center, 55131 Mainz, Germany

[3]Present address: Clinic for Neuropathlogy, Medical School, RWTH Aachen University, 52074 Aachen, Germany (HG); Biofrontera Bioscience GmbH, 51377 Leverkusen, Germany (JH)

[#]These authors contributed equally to this work.

*Correspondence should be addressed to J.H. (jhartkamp@gmx.de).

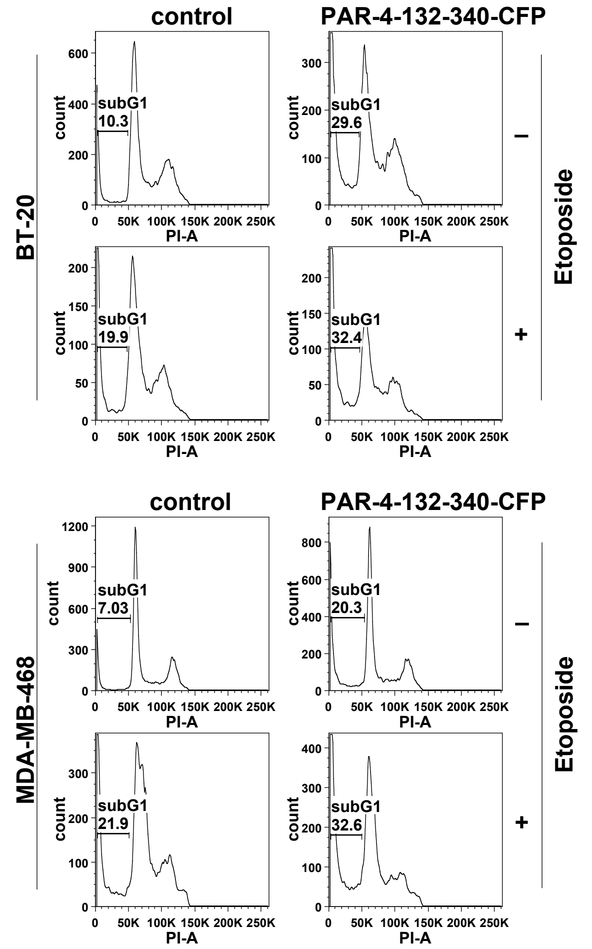

Supplementary Figure S1: Histogram belonging to Figure 4c.

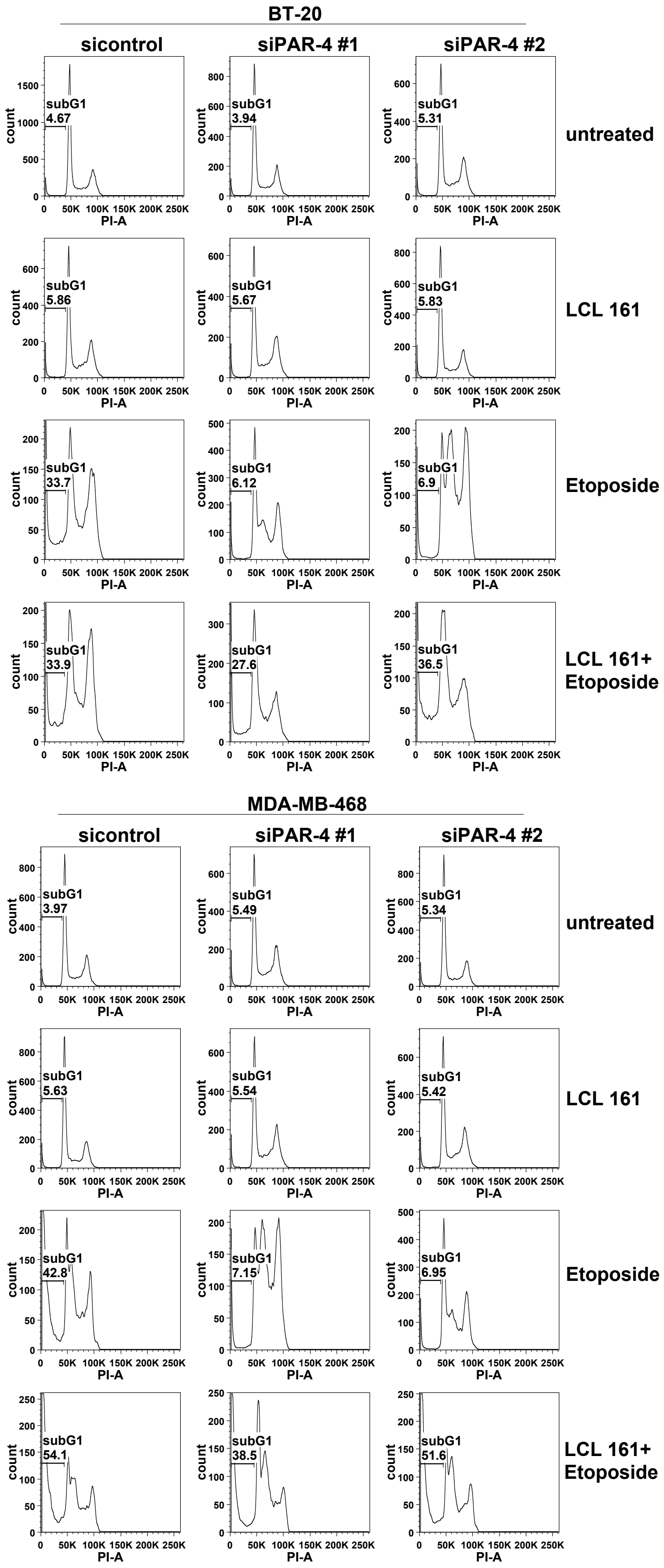

Supplementary Figure S2: Histogram belonging to Figure 6b.

Belong to Figure 1a

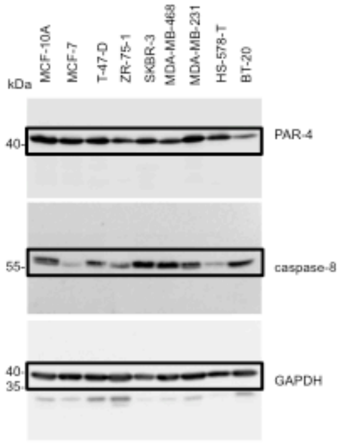

Belong to Figure 1b

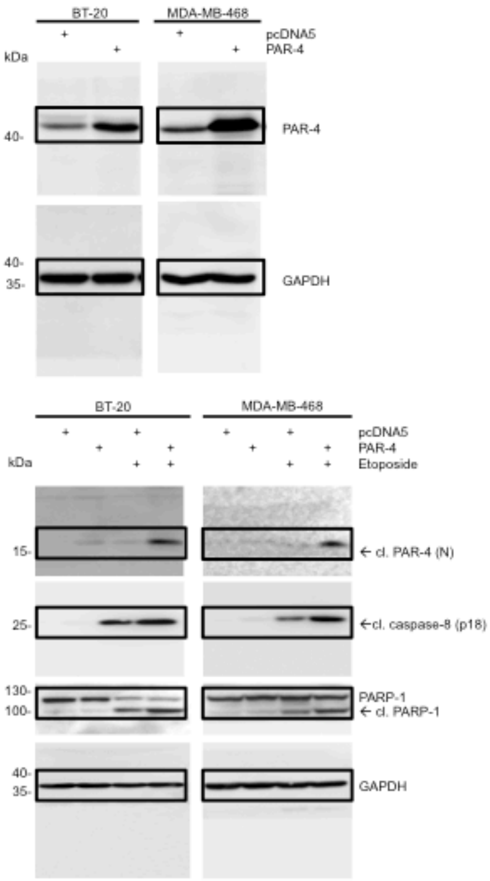

Belong to Figure 2a

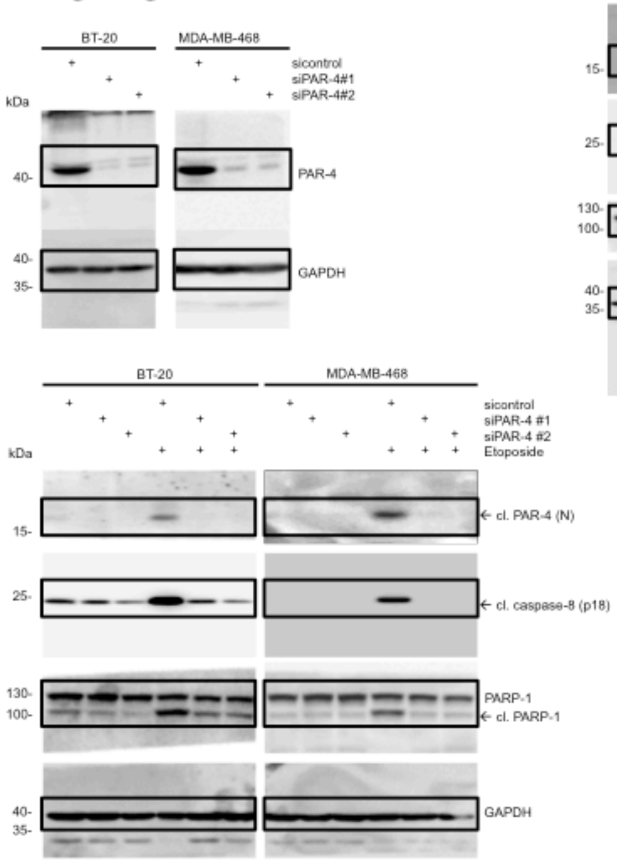

Supplementary Figure S3:

Uncropped original images of Western blots are shown belonging to Figure 1 and 2.

Belong to Figure 3a

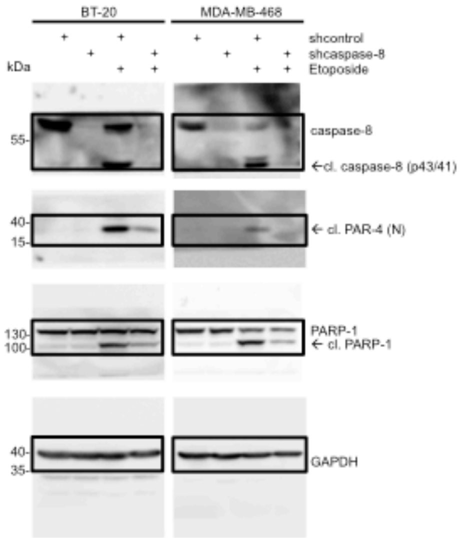

Belong to Figure 3b

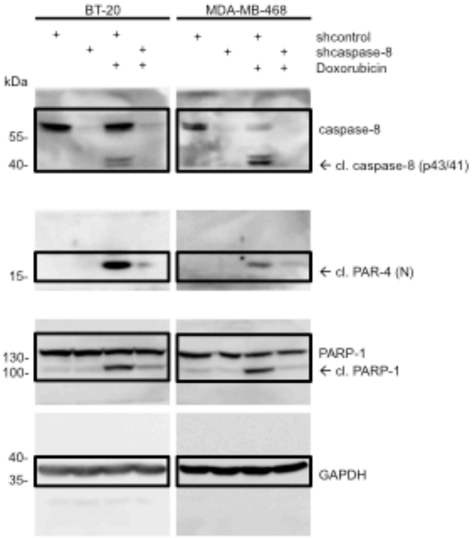

Belong to Figure 3d

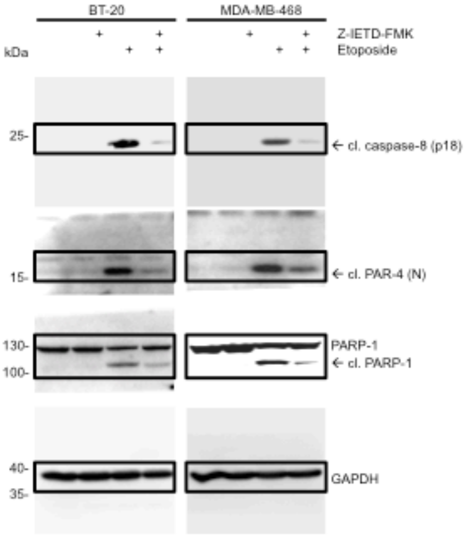

Belong to Figure 3e

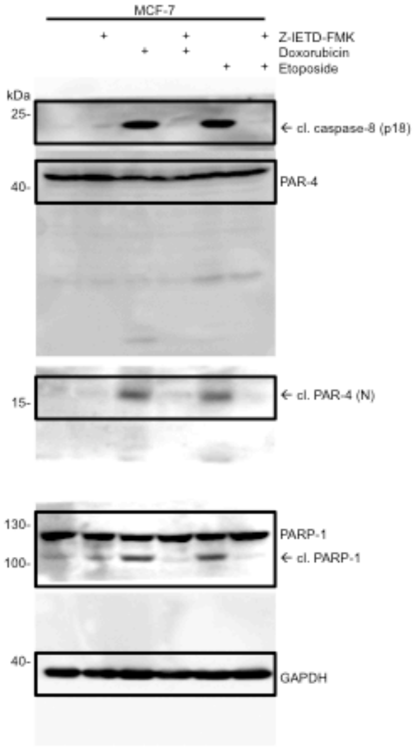

Supplementary Figure S4:

Uncropped original images of Western blots are shown belonging to Figure 3.

Belong to Figure 4b

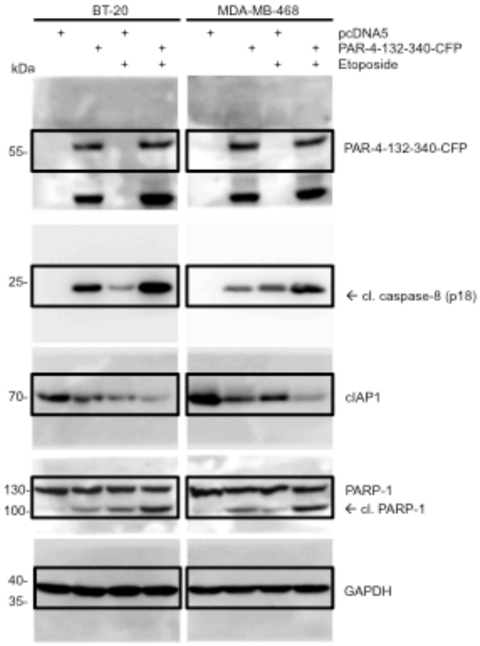

Belong to Figure 4d

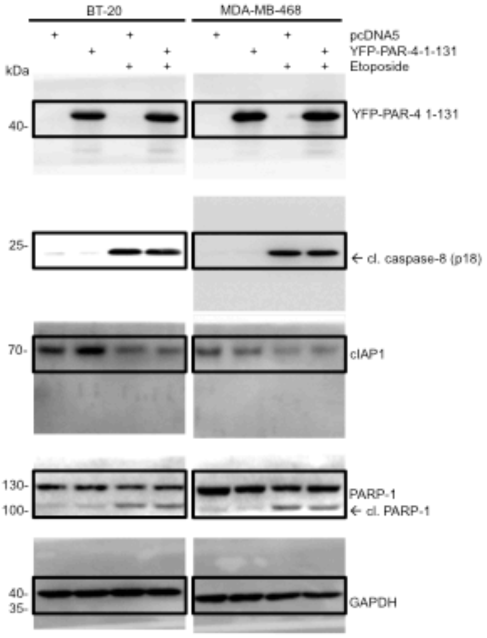

Belong to Figure 4f

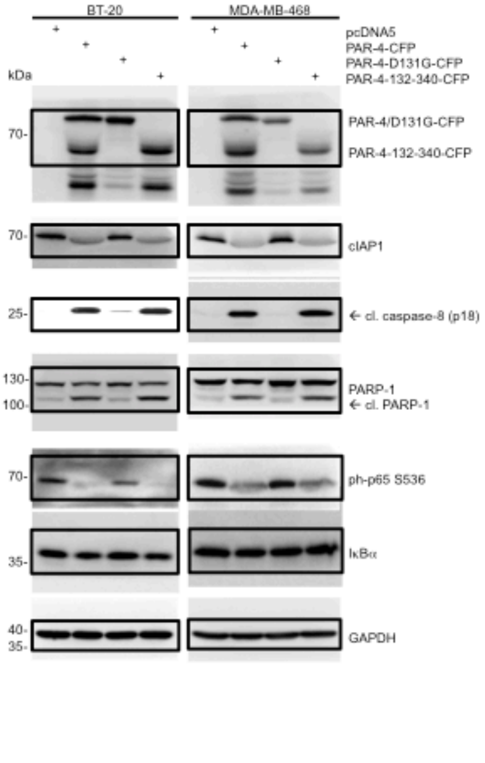

Supplementary Figure S5:

Uncropped original images of Western blots are shown belonging to Figure 4.

Belong to Figure 5a

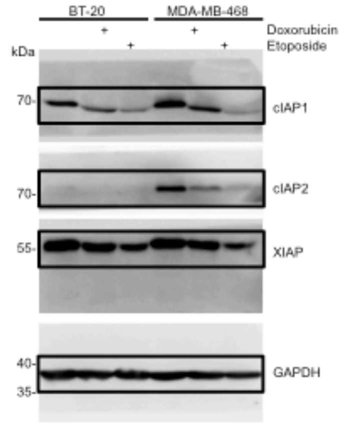

Belong to Figure 5b

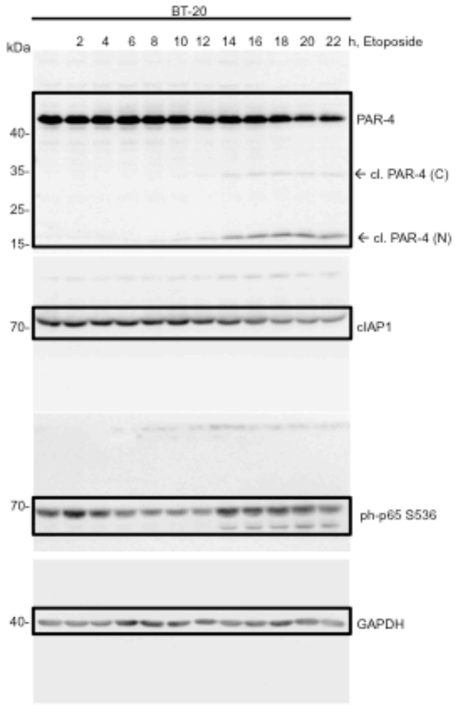

Belong to Figure 5c

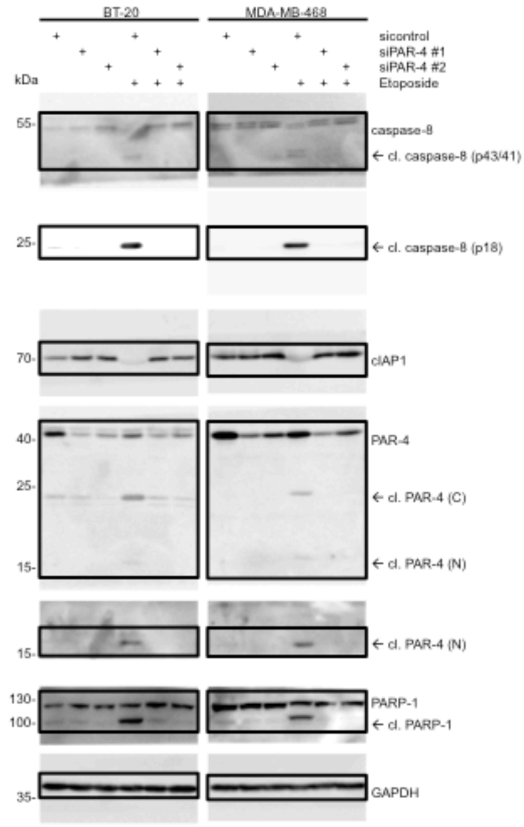

Belong to Figure 5d

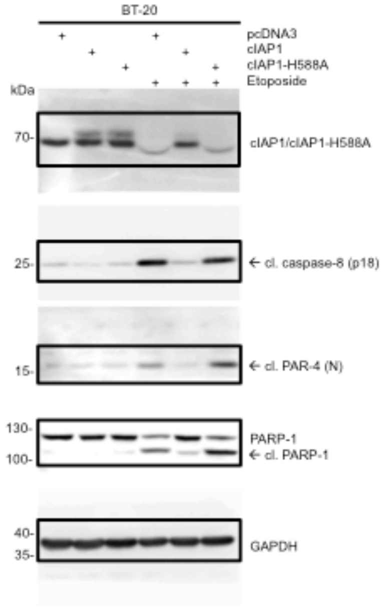

Supplementary Figure S6:

Uncropped original images of Western blots are shown belonging to Figure 5.

Belong to Figure 6a

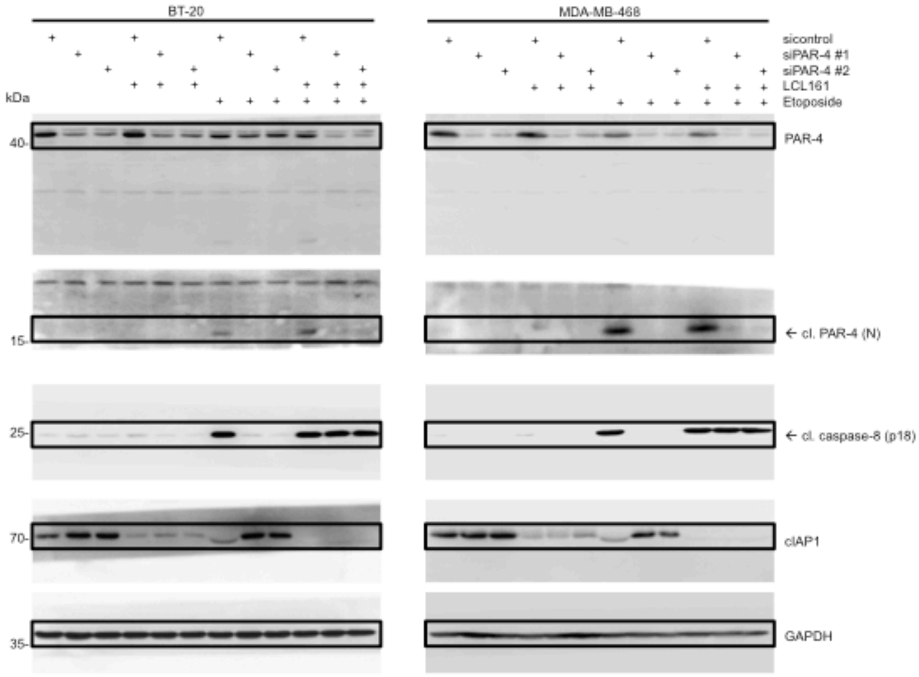

Belong to Figure 6c

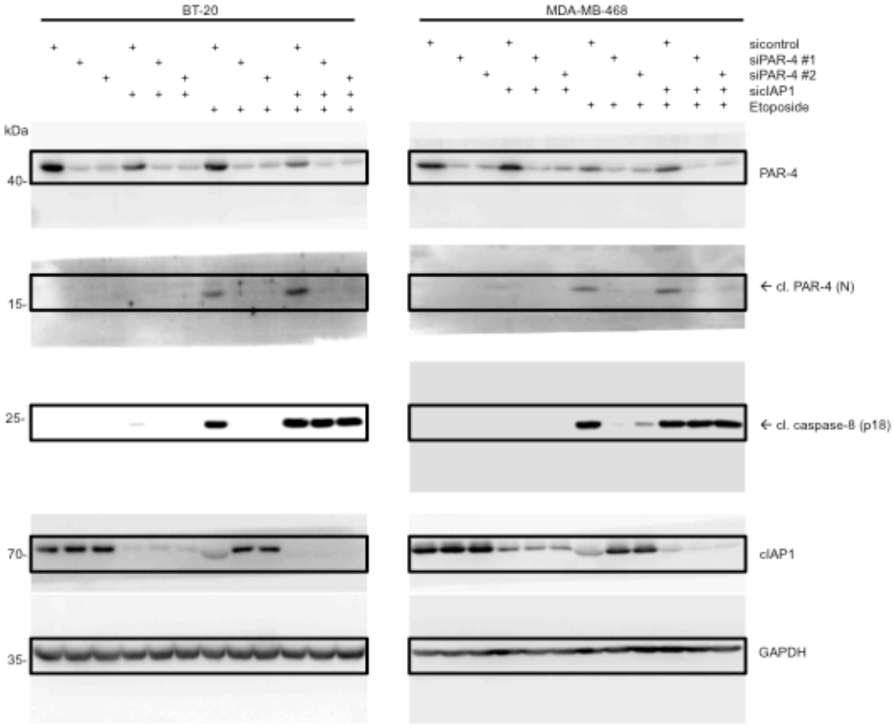

Supplementary Figure S7:

Uncropped original images of Western blots are shown belonging to Figure 6.
